# Infant, neonatal, and postneonatal mortality trends in a disaster region and in Japan, 2002–2012: a multi-attribute compositional study

**DOI:** 10.1186/s12889-019-7443-4

**Published:** 2019-08-09

**Authors:** Ai Tashiro, Honami Yoshida, Etsuji Okamoto

**Affiliations:** 1 Graduate School of Environmental Studies, Tohoku University, 468-1 Aoba, Aramaki, Aoba-ku, Sendai, Miyagi 9800845 Japan; 2 Division of Establishment for Graduate School of Health Innovation, Kanagawa University of Human Services, 2F Bldg.2-A, 3-25-10, Tonomachi, Kawasaki-ku, Kawasaki, Kanagawa 2100821 Japan; 3 Department of Health & Welfare Management, the University of Fukuchiyama, 3370, Azahori, Fukuchiyama, Kyoto, 6200000 Japan

**Keywords:** Trends in infant mortality, Neonatal/postneonatal, Causes of death, Conjoint analysis, Great East Japan earthquake and tsunami, Japan

## Abstract

**Background:**

The reductions achieved in infant mortality in Japan are globally regarded as remarkable. However, no studies in Japan have classified infant mortality trends into neonatal and postneonatal or considered regional issues. This study aimed to explore trends in neonatal and postneonatal deaths, both overall for Japan and in a region affected by a natural disaster.

**Methods:**

Drawing on national infant death data, we used a multi-attribute compositional study design to examine all infant deaths occurring in a region affected by a disaster (Tohoku, which consists of Iwate, Miyagi, and Fukushima) between 2002 and 2012. We used conjoint analysis to clarify the associations between infant and maternal characteristics and age of infant death.

**Results:**

We obtained data of a total of 31,012 infant deaths between 2002 and 2012, which included 1450 from Tohoku. Infant mortality rates in Japan overall declined over the period but increased in 2011. There were more postneonatal (29–364 days post-birth) than neonatal (0–28 days post-birth) deaths. Infant deaths in Tohoku declined slightly overall, with a fluctuation in 2011. In Tohoku, the trends in postneonatal death rates were similar; the overall rates for males increased, but those for females decreased in 2011. We found that the cause and place of infant death differed by gender for neonatal and postneonatal deaths in both Japan in general and Tohoku. The conjoint analysis showed that most variables affected the age of postneonatal death. The factor with the largest influence on the variation in infant death age was gestational week (55.5%). A maternal gestational week ≤36 was linked to an average age at death of 43.4 days, and > 37 was linked to an average of 83.7 days.

**Conclusions:**

In Japan, infant death rates have declined steadily over the past 10 years. The recent trends indicated that postneonatal death rates were higher than neonatal rates, especially in Tohoku. However, not much attention has been focused on postneonatal deaths in Japan. Our findings may help health planners to prioritise work on the factors that are linked to infant deaths in the neonatal and postneonatal periods.

**Trial registration:**

Not applicable.

## Background

Infant mortality is considered a sensitive measure of the overall health of a population, although its precise interpretation has been debated. Subnational regional disparities related to infant mortality have been studied globally [1]. Japan’s infant mortality in the pre-war period was equal to or higher than that of most of today’s developing countries, where reducing infant mortality is an urgent challenge [2]. However, infant mortality rates in Japan have declined continuously since the 1990s, in line with the contributing factors of improvements in medical advancements, such as the universal use of the Boshi Kenko Techo (maternal-child health handbook), and in paediatric facilities and medical institutions [3, 4]. In 2019, the infant mortality rate in Japan dropped to 1.96 per 1000 live births, which was the second lowest in the world (227 out of 228 countries; range: 1.85–106.3, average: 21.6 per 1000) [5].

However, such achievements in improving defunct facilities and institutions were reduced to a poor physical infrastructure in 2011 [6–8]; on 11 March 2011, a magnitude 9.0 earthquake and a tsunami struck northeast Japan. In 2011, 162 infants died in the disaster areas (Iwate, Miyagi, and Fukushima prefectures) [9]. Studies assessing the health effects of this event on the infants are ongoing. Even though scholars have long debated that a poor physical infrastructure and public health infrastructure with weak governance are a primary cause of infant mortality [10, 11], Japan has not utilised previous disaster experiences and responses, such as Hurricane Katrina, Sumatra, and the Christchurch earthquake, as lessons for evaluating and developing effective measures [12, 13].

As such, an understanding of the patterns of human casualties, which is developed over a long period, is required for disaster mitigation and management plans. However, only a few studies have attempted to quantify the impact of infant mortality in the aftermath of natural disasters over a long duration. Pregnant women and infants have unique health concerns following a natural disaster [11], but the few previous studies did not provide robust evidence for the macro trends of the mortality patterns of infant casualties, which is necessary for developing effective protection plans against large natural disasters [14, 15]. Thus, we suggest that the needs of pregnant women, infants, and breastfeeding mothers are addressed in order to change their mindset between the disaster preparedness (normal span) and disaster response (abnormal span).

The present study aimed to describe the infant mortality patterns in Japan and in the three most severely damaged prefectures in the Tohoku region between 2002 and 2012. It is useful to examine infant mortality in the Tohoku region to explore whether it differs from Japan as a whole and to assess the effects of regional issues. This is the first study on infant mortality that has focused on social and environmental determinants of infant mortality and suggests the need for health policies and interventions in the community.

One study [16] has suggested that perinatal mortality increased in Fukushima as a result of the Fukushima nuclear power plant accident, but few studies have examined infant deaths using the causes of death or a framework of the period survived; that is, neonatal (0–28 days at death) and postneonatal (29–364 days at death). Postneonatal death rates have exceeded neonatal rates across Japan, which is not consistent with an earlier study. Ogasawara and Kobayashi [2, 17] studied trends in infant mortality in Japan in 2002 according to the neonatal and postneonatal periods but did not examine factors related to gender or region. Regarding these factors, we examined whether the regional characteristics that may reduce the risk of infant mortality vary widely between disaster-affected areas and the rest of the country and in populations within the country. Thus, this study contributes to providing insights into infant mortality reduction during normal as well as abnormal periods, such as in the event of future earthquakes or other natural disasters.

## Methods

### Aim, study design, and setting

This study aimed to explore trends in neonatal and postneonatal deaths, both overall for Japan and in a region affected by a natural disaster, and to examine the association between infant death age and related characteristics.

This study used a factor control study design that included all infant deaths in the Tohoku region (Iwate, Miyagi, and Fukushima) and in Japan between 2002 and 2012. The definition of infant death used was the death of a child in the first year of life (0–364 days of age). Infant deaths were further classified into neonatal (0–28 days of age) and postneonatal (29–364 days of age).

### Data collection

We obtained data of 31,012 infant deaths from the official census forms in Japan [18]. The death forms included all women and their infants in Japan and were approved by the Ministry of Health, Labour, and Welfare (MHLW) [9, 19]. The forms were prepared by municipal governments based on death notifications and sent to the ministry via the prefecture. The items on the death forms were kept in written logs. The materials received for this report covered each calendar year included in this survey. The written logs contained notification of foetal death, including the prefectural code (1–47), gender, delivery date, foetal death date, maternal age, number of previous pregnancies, number of gestational weeks, birth weight, location of death (hospital, clinic, midwifery home, home, other), single or multiple birth, and cause of death. Following translation, causes of death were coded to match the 10th Revision of the International Classification of Diseases (ICD-10) [20].

### Infant death rates

Trends in the annual death rates (overall, by gender, and for the Tohoku region) were examined in the groups of all infants, neonates, and postneonates [18]. The denominator data were based on official annual statistics reports that included all prefectural birth rates and infant mortality rates for 2002–2012 [20, 21]. The birth data were obtained from vital statistics from the MHLW to calculate infant mortality. The annual death rates were used for the period. The neonatal and postneonatal mortality rates were compared by gender and region. The Tohoku region was considered to include Iwate, Miyagi, and Fukushima, and it was compared to all of Japan.

### Study sample and statistical analysis

The analysis included all of the infant death data in Japan for the period of 2002–2012. The leading causes of mortality (overall and by gender) were examined for both neonatal and postneonatal deaths.

Metric conjoint analysis [21, 22] was used to identify the factors that were most strongly linked to the age of infant death. To explore potential heterogeneity in the infant deaths, we fitted a regression model for infant death age so that every infant had a unique set of regression coefficients or utility scores. A utility score characterizes the relative importance of each attribute. The importance of an attribute for the preference change results from the utility range of several values of the attribute. It can be interpreted as how much difference each attribute could make in the total utility of a value. That difference is the range in the attribute’s utility values. To examine the associations between age of infant death and infant and maternal characteristics, we included interaction terms, a dummy variable, and each of the seven individual variables in the regression models.

Six individual variables were categorised as either 1 or 0 in the dataset. These included infant gender (males = 1, females = 0), gestational weeks at delivery (22–36 weeks = 1, 37–42 weeks = 0), and birth weight (< 2500 g = 1, ≥ 2500 g = 0). In addition, women whose first infant died were defined as primiparous (0) and those who had given birth before as multiparous (1). Maternal age was categorised into two groups: 15–34 (1) and 35 years or older (0). We used a dummy variable to indicate whether there had been an earthquake with an intensity of over six and a magnitude of at least six at least once during the year in which the child was born (occurred in the year = 1, none = 0) between 2002 and 2012. The regional domain variable was given a value of 1 for Tohoku and 0 for other areas. The exposure variable was the infant death age (less than 1 year, 0–364 days old).

Metric conjoint analysis models use a simple main-effects analysis of variance (ANOVA) with some specialised output. This model was used to perform a trend analysis of the infant death factors over time. The attributes were the independent variables, the judgements were the dependent variable, and the part-worth utilities were the βs, which were the parameter estimates from the ANOVA model. The following formula shows the model for the six variables:

1 $$ {y}_i=\mu +{\beta}_{1i}+{\beta}_{2i}+{\beta}_{3i}+{\beta}_{4i}+{\beta}_{5i}+{\beta}_{6i}+{\varepsilon}_{i.} $$

where,

2 $$ \sum {\beta}_{1i}=\sum {\beta}_{2i}=\sum {\beta}_{3i}=\sum {\beta}_{4i}=\sum {\beta}_5i=\sum {\beta}_{6i}=0. $$

The *y*_*i*_ term is the subject’s infant death age with *i* and the dummy of each variable (*i* = 0, 1). The mean is *μ*, and the error is *ɛ*_*i*_. The predicted utility for the *i* product is:

3 $$ {\hat{y}}_i=\hat{\upmu}+{\hat{\beta}}_{1i}+{\hat{\beta}}_{2i}+{\hat{\beta}}_{3i}+{\hat{\beta}}_{4i}+{\hat{\beta}}_{5i}+{\hat{\beta}}_{6\mathrm{i}.} $$

The *R*-square increases during every iteration until convergence, when the change in *R*-square is essentially zero. The *R*-square for a nonmetric conjoint analysis model is always greater than or equal to the *R*-square from a metric analysis of the same data. A smaller *R*-square in the metric conjoint analysis is not necessarily a disadvantage since the results should be more stable and reproducible with the metric model [23]. We computed these equations using Stata 14.1 [23]. To compare the contribution of the six attributes, the relative importance of each was computed using the following steps: (i) the multicollinearity was examined using a variance inflation factor (VIF) to observe if this factor was around 1.0, (ii) the collation among the independent variables was checked, (iii) the total range was derived, and (iv) the relative importance of each attribute was calculated. All analyses were performed with a significance level of *p* <  0.01.

## Results

### Trends in annual death rates by region

**Fig. 1:**
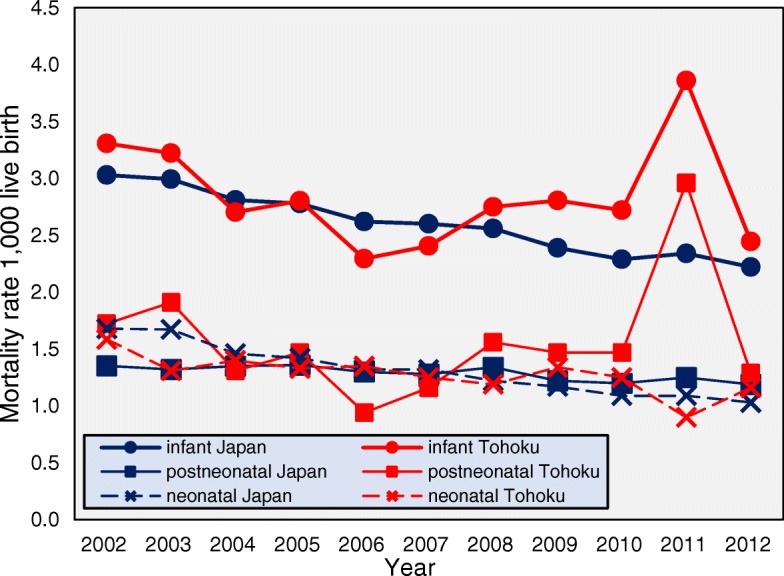
Infant, neonatal, and postneonatal mortality rates for Japan and Tohoku, 2002–2012. *Note*. Infant (< 1 year), neonatal (0–28 days of age), and postneonatal (29–364 days of age). The *p*-value is based on a *t*-test. *p* < 0.001

Between 2002 and 2012, there were 31,012 infant deaths in Japan and 1450 in Tohoku. The trends in the infant, neonatal, and postneonatal mortality rates for Japan and Tohoku during this period are shown in Fig. 1.

The overall infant mortality rates declined over the period. The postneonatal mortality rate exceeded the neonatal mortality rate from 2006 onwards. In Tohoku, however, the infant mortality rate fluctuated across the period and was higher than in Japan except in 2004, 2006, and 2007. In 2011, the rate in Tohoku was 1.6 times higher than that in Japan (3.9 vs. 2.3, *p* <  0.001). The postneonatal mortality rate was 3.3 times higher than the neonatal rate (3.0 vs. 0.9, *p* <  0.001) in Tohoku. The Great East Japan Earthquake and Tsunami (GEJET) seems to have had a greater effect on postneonatal deaths than on neonatal deaths. In 2012, there were no major differences in the death rates among neonates and postneonates in either Tohoku or Japan.

### Trends in the annual death rates by gender

**Fig. 2:**
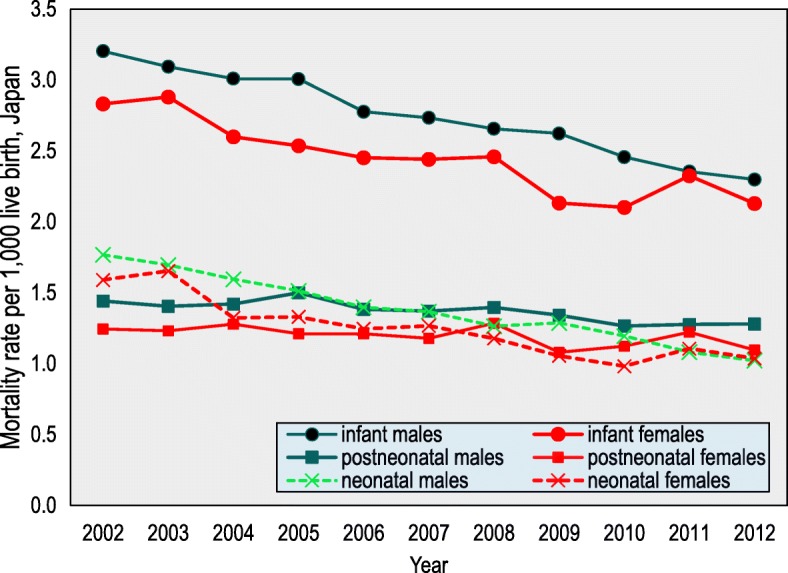
Infant, neonatal, and postneonatal mortality rates by age for Japan, 2002–2012. *Note*. Infant (< 1 year), neonatal (0–28 days of age), and postneonatal (29–364 days of age). The *p*-value is based on a *t*-test. *p* < 0.001

Figure 2 shows the trends in infant, neonatal, and postneonatal deaths by gender in Japan. The overall trend was that the infant, neonatal, and postneonatal rates by gender declined over the period. While the postneonatal male mortality rate exceeded the rate for neonates in 2007, neonatal mortality rates in both males and females continued to decline. The postneonatal mortality rates fluctuated, but the mortality rates by gender were all similar in 2012, except for the rate for postneonatal males. The neonatal male mortality rate was highest in 2002 and the rates among females also declined (*p* <  0.001).

**Fig. 3:**
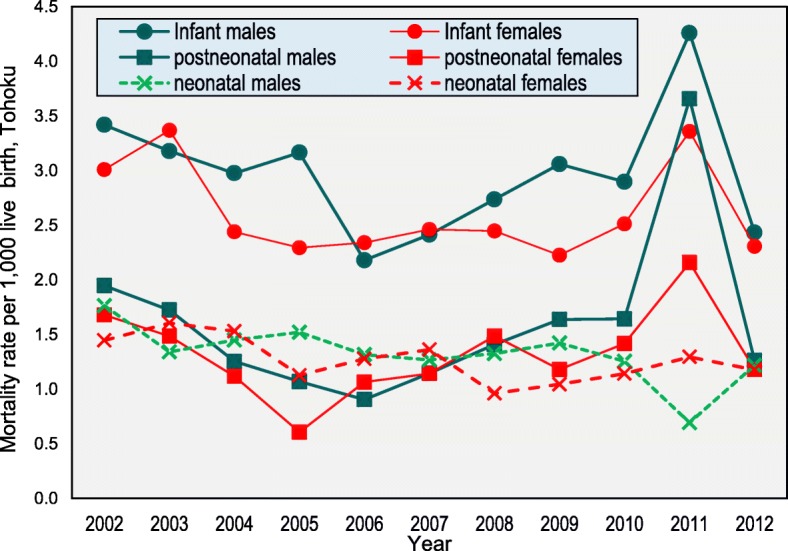
Infant, neonatal, and postneonatal mortality rates by age for Tohoku, 2002–2012. *Note*. Infant (< 1 year), neonatal (0–28 days of age), and postneonatal (29–364 days of age). The *p*-value is based on a *t*-test. *p* < 0.001

Figure 3 shows the annual trends in the infant, neonatal, and postneonatal death rates by gender in the Tohoku region. These rates generally fluctuated. Unlike the death rate trends in Japan (Fig. 2), the postneonatal death rates for both genders were higher than the neonatal death rates during the period. The postneonatal death rates were higher for males than females in 2011, although the shape of the curves was similar.

### Causes of death

Between 2002 and 2012, there were 31,021 infant deaths in Japan and 1450 in Tohoku. The top five causes of infant death are shown in Table 1. Across Japan, for male infants, the most common cause was sudden infant death syndrome (SIDS) (6.4%), followed by ill-defined or unknown cause (4.4%), trisomy (3.8%), congenital hypoplasia/dysplasia of the lung (2.9%), and hypoplastic left heart syndrome (2.4%). For female infants, the leading causes of death were trisomy (6.9%), SIDS (5.2%), ill-defined or unknown cause (3.6%), congenital hypoplasia/dysplasia of the lung (2.5%), and extremely low birth weight (2.4%). Table 1 shows that in Tohoku, trisomy, asphyxia, and extremely low birth weight were common causes of death in both genders.

**Table 1:** Infant deaths (< 1 year) in Tohoku and Japan, 2002–2012, by cause and gender

Japan								Tohoku							
Males				Females				Males				Females			
Rank	Category (ICD)	Freq.	%	Rank	Category (ICD)	Freq.	%	Rank	Category (ICD)	Freq.	%	Rank	Category (ICD)	Freq.	%
1	SIDS	1068	6.4	1	Trisomy 18	986	6.9	1	SIDS	61	7.9	1	SIDS	38	5.8
2	Ill-defined and unknown cause of mortality	742	4.4	2	SIDS	740	5.2	2	Unspecified effects of drowning and nonfatal submersion	39	5.0	2	Trisomy 18	32	4.9
3	Trisomy 18	639	3.8	3	Ill-defined and unknown cause of mortality	512	3.6	3	Extremely low birth weight newborn	23	3.0	3	Severe neonatal asphyxia	19	2.9
4	Congenital hypoplasia and dysplasia of the lung	494	2.9	4	Congenital hypoplasia and dysplasia of the lung	360	2.5	4	Asphyxia	23	3.0	4	Congenital hypoplasia and dysplasia of the lung	19	2.9
5	Hypoplastic left heart syndrome	404	2.4	5	Extremely low birth weight newborn	346	2.4	5	Trisomy 18	22	2.8	5	Ill-defined and unknown cause of mortality	19	2.9
	All other	13,460	80.1		All other	11,270	79.3		All other	609	78.4		All other	527	80.6
	Total	16,807	100.0		Total	14,214	100.0		Total	777	100		Total	654	100.0

*Note.* *ICD* International Classification of Diseases, *SIDS* sudden infant death syndrome

Table 2 shows that the causes of death for neonates differed by gender and across the two regions. SIDS was not in the top five causes of death for neonates of either gender; instead, congenital hypoplasia, trisomy, low birth weight, and severe neonatal asphyxia were the main causes of death. SIDS was the most common cause of death among postneonates across all genders and regions (Table 3). The other main causes of postneonatal death were trisomy, ill-defined or unknown cause, and asphyxia.

**Table 2:** Neonatal deaths (0–28 days) in Japan and Tohoku, 2002–2012, by cause and gender

Japan								Tohoku							
Males				Females				Males				Females			
Rank	Category (ICD)	Freq.	%	Rank	Category (ICD)	Freq.	%	Rank	Category (ICD)	Freq.	%	Rank	Category (ICD)	Freq.	%
1	Congenital hypoplasia and dysplasia of the lung	446	5.1	1	Trisomy 18	434	5.8	1	Extremely low birth weight newborn	19	5.4	1	Severe neonatal asphyxia	19	5.9
2	Trisomy 18	390	4.5	2	Congenital hypoplasia and dysplasia of the lung	327	4.4	2	Bacterial sepsis of newborn, unspecified	17	4.8	2	Congenital hypoplasia and dysplasia of the lung	17	5.3
3	Severe neonatal asphyxia	354	4.1	3	Extremely low birth weight newborn	326	4.3	3	Severe neonatal asphyxia	15	4.2	3	Primary atelectasis of newborn	13	4.0
4	Extremely low birth weight newborn	348	4.0	4	Primary atelectasis of newborn	302	4.0	4	Congenital hypoplasia and dysplasia of the lung	15	4.2	4	Trisomy 18	13	4.0
5	Primary atelectasis of newborn	331	3.8	5	Severe neonatal asphyxia	299	4.0	5	Birth-related asphyxia	13	3.7	5	Extremely low birth weight newborn	10	3.1
	All other	6823	78.5		All other	5808	77.5		All other	276	77.7		All other	250	77.6
	Total	8692	100.0		Total	7496	100.0		Total	355	100.0		Total	322	100.0

*Note*. *ICD* International Classification of Diseases

**Table 3:** Postneonatal deaths (29–364 days) in Japan and Tohoku, 2002–2012, by cause and gender

Japan								Tohoku							
Males				Females				Males				Females			
Rank	Category (ICD)	Freq.	%	Rank	Category (ICD)	Freq.	%	Rank	Category (ICD)	Freq.	**%**	Rank	Category (ICD)	Freq.	%
1	SIDS	980	12.1	1	SIDS	671	9.9	1	SIDS	51	12.1	1	SIDS	35	10.5
2	Ill-defined and unknown cause of mortality	696	8.6	2	Trisomy 18	552	8.2	2	Unspecified effects of drowning and nonfatal submersion	34	8.1	2	Trisomy 18	19	5.7
3	Asphyxia	338	4.2	3	Ill-defined and unknown cause of mortality	462	6.9	3	Asphyxia	19	4.5	3	Ill-defined and unknown cause of mortality	19	5.7
4	Trisomy 18	249	3.1	4	Asphyxiation	242	3.6	4	Ill-defined and unknown cause of mortality	18	4.3	4	Unspecified injury	15	4.5
5	Foreign body in respiratory tract, part unspecified	223	2.7	5	Ventricular septal defect	212	3.2	5	Trisomy 18	12	2.8	5	Ventricular septal defect	13	3.9
	All other	5629	69.4		All other	4579	68.2		All other	288	68.2		All other	231	69.5
	Total	8115	100.0		Total	6718	100.0		Total	422	100.0		Total	332	100.0

*Note*. *ICD* International Classification of Disease, *SIDS* sudden infant death syndrome

### Place of death

**Fig. 4:**
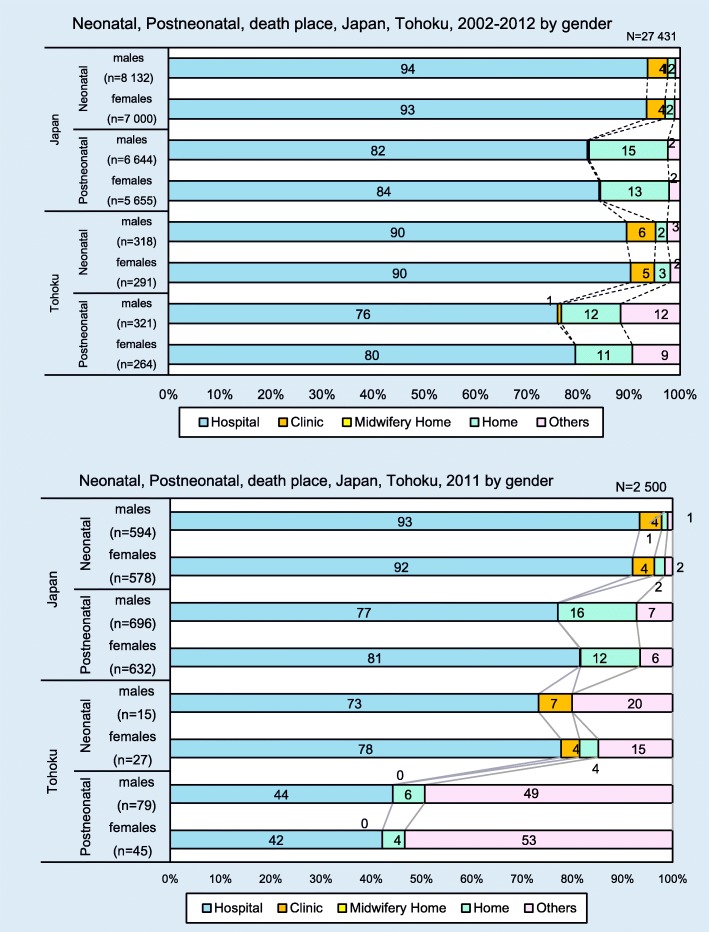
Neonatal and postneonatal place of death (Japan and Tohoku) by gender (2002–2012 and 2011)

Figure 4 shows the place of neonatal and postneonatal death in Japan and Tohoku by gender between 2002 and 2012 as well as in 2011, specifically. Between 2002 and 2012, we obtained data about the place of death for 27,431 infants in Japan and 1431 in Tohoku. More than 75% of infants died in hospital in both Japan and Tohoku. More postneonates than neonates died at home. The percentage of postneonates who died in other places in Tohoku was higher than for neonates in Tohoku or neonates and postneonates in Japan. For 2011, we collected data about the place of death of 2500 neonates, which included 166 in Tohoku. In Japan, over 90% of these children died in hospital, with the second largest group dying at home. In Tohoku, around 75% of neonates died in hospital (males = 73%, females = 78%), but around 50% of postneonates who were affected by GEJET died in other places (males = 44%, females = 42%).

### Characteristics related to the age of death of the infants

Table 4 shows the characteristics of the infants and their mothers according to the results of the conjoint analysis. Any data indicating over 43 weeks of gestation or where the weeks of gestation were unknown were removed from the dataset. The total number of observations was 27,002 in this model. The average VIF for each variable was 1.23 (1.01–1.65). There were no interaction variables. All six variables were, therefore, applied in this model with a standard significance level set at 5%.

**Table 4:** Attribution of the factors to the infant death age

Infant mortality age (0–364 days)					
Variables	Utility	95%CI	Coef.	*SE*	*p*
Gender					
Male	62.2	60.8, 63.6	−2.38	1.05	0.023
Female	64.6	63.1, 66.1			
Birth weight					
0–2499 g	58.5	57.1, 60.0	−12.4	1.38	< 0.001
2500 g ≤	70.9	68.9, 72.9			
Mother’s age					
15–34 years	62.5	62.4, 64.8			
35 years ≤	63.6	60.5, 64.5	−1.16	1.19	0.33
Gestational week					
22–36 weeks	43.4	41.7, 45.0	−40.28	1.34	< 0.001
37–42 weeks	83.7	81.9, 85.3			
Parity					
Primiparous	55.9	54.4, 57.5	−13.1	1.06	< 0.001
Multiparous	69.1	67.7, 70.5			
Mega earthquake					
Intensity 6 <	62.3	61.0, 63.6	−2.78	1.08	0.001
Intensity ≤6	65.1	63.4, 66.8			
Region					
Tohoku	63.8	58.9, 68.7	0.51	2.57	0.842
Other regions	63.3	62.2, 64.3			

*Note*. *p* < 0.000, *R*-squared = 0.81, Adj. *R*-squared = 0.82

Utility indicates average importance value on infant death age

*SE* = standard error; Coef. = coefficient. Statistically significant at *p* < 0.05

*Source*. Calculated by the authors from the data of the national infant death forms from the Ministry of Health, Labour, and Welfare

Utility indicates average importance value on infant death age. These values provide a measure of the relative importance of the single factors for the determination of the utilities. The utility mean of the female infant death age was 2.4 days higher than that of males (64.6, 95% confidence interval [CI]: 63.1–66.1 vs. 62.2, 95% CI: 60.8–63.6). Infants of birth weight < 2499 g were on average 58.5 days old at death (95% CI: 57.1–60.0), and those of 2500 g or more were on average 70.9 days old (95% CI: 68.9–72.9).

Infants in the dataset whose gestational week was 37–42 (83.7, 95% CI: 81.9–85.3) lived on average 40.3 days longer than those whose gestational week was 22–36 (43.4, 95% CI: 41.7–45.0). Maternal primiparity (55.9, 95% CI: 54.4–57.5) was associated with an earlier age of death—on average, 13.0 days sooner than infants of multiparous mothers (69.1, 95% CI: 67.7–70.5). An earthquake of an intensity of less than six was associated with an average age at death 2.78 days later than one of greater intensity (65.1, 95% CI: 63.4–66.8 vs. 62.3, 95% CI: 61.0–66.8). The mothers’ age and region were not statistically significantly different (*p* > 0.05).

**Fig. 5:**
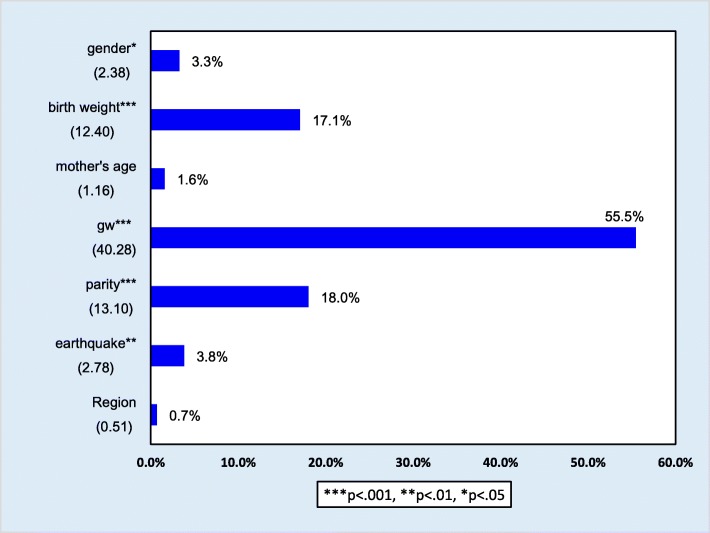
Percentage of variation in age at death that can be attributed to the characteristics. ***Note.*** gw = gestational week

Figure 5 presents the percentage of variation in age at infant death that can be attributed to particular characteristics (statistical significance level *p* <  0.05). The percentages indicate attribute importance values on infant death age. The higher the percentage, the more important factor in contributing to infant death age. As shown in this figure, 55.5% of the variation in age at infant death was most strongly attributed to gestational week (*p* <  0.001). The second most important factor was parity (18.0%, *p* < 0.001) and the third was birth weight (17.1%, *p* < 0.01). The contribution of a large earthquake during the year was much smaller (3.8%, *p* < 0.01), as was gender (3.3%, *p* < 0.05).

## Discussion

To our knowledge, this is the first standardised statistics-based study of infant mortality with an official total sample size that has focused on the association between the infant death age and particular characteristics and that has covered both Japan in general and Tohoku. The infant, neonatal, and postneonatal mortality rates all gradually decreased in Japan from 2002 to 2012. In contrast, these rates in Tohoku region fluctuated. While the postneonatal mortality rates decreased during the period from 2002 to 2012 in Japan, mortality in the Tohoku region has increased since 2006 (Fig. 1, Fig. 2). Although it is proposed that postneonatal mortality is preventable, this study showed that the postneonatal death rates exceeded the neonatal death rates across Japan, which is not consistent with an earlier study [24–26]. One possible factor for this increase is GEJET and its associated events. Generally, rural areas have higher infant mortality than urban areas [27]. GEJET cased in Tohoku region where is a rural region in Japan. Moreover, Japan has only recently achieved the world’s highest medical standard for mothers and infants at an early stage, so appropriate measures for disaster response and prevention for neonates and also postneonates did not exist at the time of the disaster. Hence, this oversight of the need to establish appropriate measures increased postneonatal death during the emergency in 2011. Even when obstetricians and gynaecologists, midwives, and paediatricians were dispatched to the disaster-affected areas, there were many isolated mothers and infants who were unable to receive adequate care [27]. Even if the perinatal facilities resumed in the afflicted area, some pregnant women and mothers with infants were not able to receive a newborn medical check-up in the afflicted area in 2011 [28].

We also found that the cause and place of infant deaths differed by gender and among neonates and postneonates and that such trends in the neonatal and postneonatal mortality rates changed in the last decade. Compared to Japan, more postneonates in Tohoku died outside of hospitals. This trend was remarkable in 2011. From these results, we can propose that it is necessary to provide appropriate care (e.g. access to healthcare, information, and maternal physical support that considers the vulnerability of the healthcare structure or health infrastructure) for postneonates in Tohoku during both a normal and an abnormal span.

In this study, we ranked the attribution of several infant and maternal characteristics to the variation in the age of infant death using conjoint analysis. The factor that made the most substantial contribution to the variation in age of infant death was the gestational week (55.5%). We found that a maternal gestational week of 36 or less was linked to an average age at death of 43.4 days, compared with 83.7 days for a maternal gestational week of 37 or later. This was the most extended death age in this model. Previous studies have suggested that the earlier the gestational week, the higher the infant’s risk of death [28]. In terms of the biological mechanism, Matheus et al. reported that foetal development and gestational age had a linear correlation up to 34 gestational weeks [29]. They also suggested that foetal growth progresses rapidly between 20 and 38 gestational weeks. To expand related matters, Crump et al. suggested that early gestational age increases mortality in early childhood and young adulthood [30]. Considering these studies, the results of the present study suggest that birth during rapid progression would accelerate the risk of early death.

Low birth weight (under 2400 g) was the second largest contributor to a later date of death (70.9 days). Previous research has highlighted the effect of advanced maternal age on increasing the risk of infant death [15], but this factor was not statistically significant in our model (*p* < 0.05). Earthquakes were a stronger contributor to the variation in the age of infant death.

There have been several health promotion activities and action plans for the prevention of infant deaths in Japan; however, most have not focused on postneonatal deaths, and there is no framework for the prevention of postneonatal deaths. The increases in both the male and female postneonatal death rates were remarkable in the Tohoku region, which is characterised as a vulnerable earthquake disaster area.

In Japan as a whole, the infant death rates decreased in Tohoku across the period examined for this study, although there seem to have been few practical measures in neonatal/postneonatal care to improve the preparedness for natural disasters. While neonatal deaths are generally associated with the pregnancy and delivery periods, postneonatal deaths are more reflective of maternal physical condition and social conditions [31, 32]. In 2011, the number of postneonatal deaths outside of hospitals was higher than that of the other places of death in Tohoku. This result shows that it is necessary to pay more attention to differences in regional infant mortality trends and the effects of disasters. To reduce infant mortality, it is essential to develop supportive health policies and a community healthcare system for mothers. The healthcare system needs to provide appropriate care for newborns and infants, access to healthcare, and a support system for maternal health.

This study had several limitations. First, it covered only infant deaths. We were not able to compare this with infants who survived or to identify the odds ratio or relative risk among those who died or survived. The study also focused on Tohoku, which is an area that has been severely affected by earthquakes, particularly the GEJET disaster event. Further study is needed to compare this region and Japan as a whole with other regions that experience natural disasters. However, to the best of our knowledge, this is the first study to examine the cause of infant death in both the neonatal and postneonatal periods, and it has identified new age at death trends that are related to several maternal and infant characteristics in one particular region.

## Conclusions

In Japan, the infant death rates declined steadily between 2002 and 2012, but the postneonatal death rates have recently become higher than the neonatal death rates, especially in a disaster-hit region. Little attention has been paid to postneonatal deaths in Japan, and most postneonatal deaths are preventable. Depicting regional differences in infant mortality as well as access to services and the coverage of interventions represents a powerful approach for assessing the inequities. Investigating the relationship between infant mortality trends and subnational or regional administrative units is critically important for understanding more proximal determinants and policy responses to the disparities [15]. Infant mortality reflects interactions between several regional factors, including the neonatal and postneonatal care in different locations, the vulnerabilities of public health systems, and the effect of natural disasters. Health planners should, therefore, draw on our study’s findings to prioritise actions to resolve factors that are linked to age at infant death. Public health decision-makers should also consider regional characteristics when allocating medical and public health resources for both neonatal and postneonatal care.

## Data Availability

The datasets supporting the conclusions of this article are officially available in the Infant Mortality of Vital Statistics Survey Death Form from the Ministry of Health, Labor and Welfare (MHLW) according to the Statistics Act (Act No. 53 of 2007). Access to the datasets is also limited unless appropriate permissions have already been obtained. In this study, the authors ordered infant death data logged in text files, including copies of Vital Statistics Survey Death Forms on magnetic media upon approval of MHLW in the official census forms in fiscal year between 2002 and 2012. Research involving anonymized records and data sets exist in the public domain. Therefore, it is not possible to identify individuals from the information provided. Furthermore, after this study concludes, those infant death forms are to be crossed out. Hence, the authors could not share our data since doing so is strictly prohibited by the vital statistical law in Japan. When an inquiry about the dataset is needed, an application is required for MHLW (https://www.e-stat.go.jp/surveyitems/surveyforms/01201).

## Abbreviations

ANOVA: analysis of variance
CI: confidence interval
GEJET: the Great East Japan Earthquake and Tsunami
ICD-10: 10th Revision of the International Classification of Diseases
MHLW: Ministry of Health, Labour, and Welfare
SIDS: sudden infant death syndrome
VIF: variance inflation factor
